# *Candida* Isolates From Blood and Other Normally Sterile Foci From ICU Patients: Determination of Epidemiology, Antifungal Susceptibility Profile and Evaluation of Associated Risk Factors

**DOI:** 10.3389/fpubh.2021.779590

**Published:** 2021-11-11

**Authors:** Bo Wang, Xinlong He, Feng Lu, Yajuan Li, Yuerong Wang, Min Zhang, Ying Huang, Jinxing Xia

**Affiliations:** ^1^Department of Clinical Laboratory, The First Affiliated Hospital of Anhui Medical University, Hefei, China; ^2^Department of Pathogen Biology, School of Medicine, Yangzhou University, Yangzhou, China; ^3^Jiangsu Key Laboratory of Experimental and Translational Non-coding RNA Research, Yangzhou University, Yangzhou, China

**Keywords:** intensive care unit (ICU), candidiasis, risk factors, drug resistance mechanism, epidemiology

## Abstract

**Background:** The clinical diagnosis and therapy for ICU patients with invasive candidiasis are challenged by the changes of *Candida* community composition and antimicrobial resistance. The epidemiology and drug sensitivity of candidiasis in ICU as well as its risk factors and drug resistance mechanism were investigated.

**Methods:** In the present study, 115 patients in ICU were recruited from June 2019 through July 2020. Among them, 83 *Candida* isolates were identified with MALDI-TOF mass spectrometry. The susceptibility to antifungals was measured by microdilution method. The molecular mechanisms of azole-resistant *Candida tropicalis* were explored by sequencing, and their outcomes were explicitly documented.

**Results:** *Candida glabrata* and *C. tropicalis* were the predominant non-*C. albicans Candida*. The specimen sources were mainly urine, bronchoalveolar lavage fluid and blood. The age, length of hospitalization, tracheotomy, diabetes and concomitant bacterial infection were the main risk factors for candidiasis. The majority of *Candida* species exhibited susceptibility to antifungals. However, certain *C. tropicalis* were frequently resistant to azoles. The polymorphism of the *ERG11* in *C. tropicalis* was likely associated with azole resistance.

**Conclusion:** The multiple risk factors for candidiasis in ICU patients need to be considered. Certain *C. tropicalis* exhibit resistance to azoles likely due to the *ERG11* gene polymorphism.

## Introduction

*Candida* species is able to result in clinically invasive infections, commonly referred to as invasive candidiasis (IC). It can lead to skin and mucosal lesions, fungemia, and occasionally multiple focal infections. The pattern of its symptom varies with the different infectious sites (1). The infection caused by *Candida* spp. is among the top three infections commonly taking place in the intensive care units (ICUs) worldwide (2, 3), accounting for 18% of all infections (4). In particular, annually there are hundreds of thousands of patients who are inflicted with IC globally. Owing to its high mortality (about 70%), IC has been regarded as an emerging threat to public health (2). Since patients from the ICUs usually harbor impaired physiological and immune functions, probably because of the underlying diseases, clinically invasive interventions, hormone treatments, or hospitalization duration (5), they are prone to developing IC. The different *Candida* spp. differ in the severity of invasive infections, treatment strategies, and disease prognosis. As the widespread utilization of broad-spectrum antifungal agents and prophylactic empirical treatments are extensively applicable in clinic, the IC infection rate among the acute and severe patients is annually elevated. The alteration of fungal community composition and antimicrobial resistance has posed a potential threat to the clinical diagnosis and treatment of IC (6, 7). Timely and rational usage of antifungals is of great significance to the prognosis of those patients in the ICUs. Hence, it is critically essential for better early treatment and improvement of clinical outcomes of the patients to investigate the epidemiological features and to profile the antifungal sensitivity of IC in local regions.

In the present study with the associated clinical data, we evaluated the species distribution and antifungal agent sensitivities of *Candida* spp., and the clinical features and the diverse risk factors of ICU patients with IC. Moreover, the 14-α-sterol demethylase (*ERG11*) and sterol δ5,6-desaturase (*ERG3*) genes of azole-resistant *C. tropicalis* were sequenced to explore the molecular mechanism of drug resistance. Meanwhile, the therapeutic effects observed from the patients with azole-resistant *C. tropicalis* were explicitly documented in current study.

## Materials and Methods

### Patient Data Collection

The present study recruited the patients who were admitted to the ICU in a tertiary hospital of China from June 2019 through July 2020. The criteria for diagnosing IC complied with the Chinese expert consensus statement from the Chinese Medical Association (8, 9), and the revised definitions of invasive fungal disease from the European Organization for the Research and Treatment of Cancer/Mycoses Study Group consensus group (10). All the subjects' clinical and laboratory information was retrieved from the digital data system of the hospital. This study excluded the same isolate of *Candida* species that appeared in repeated cultures from the same patient. A variety of risk factors that influence the occurrence of IC were investigated, including invasive interventions or procedures, immunosuppressive status, surgery, ICU length of stay.

### Microorganism Identification and Antifungal Susceptibility

Specimens were obtained from blood, urine, catheter, and other normal sterile body fluids including drainage fluid, ascites, pleural fluid, bronchoalveolar lavage (BAL), and incision secretion of ICU patients, and were then inoculated onto plates of Sabouraud Dextrose Ágar and CHROMagar *Candida* chromogenic Agar for culture at 35°C for 48 h, except for blood samples which were directly tested on an automatic microbial identification system (BacT/ALERT 3D, bioMérieux, Marcy l'Étoile, France). *Candida* spp. were identified through matrix-assisted laser desorption ionization-time of flight mass spectrometry (MALDI-TOF MS, bioMérieux, Marcy l'Étoile, France). Antifungal susceptibility tests for fluconazole, itraconazole, voriconazole, amphotericin B and flucytosine, were performed for all *Candida* isolates using an ATB FUNGUS 3 kit (bioMérieux, La Balme-les Grottes, France). The minimal inhibitory concentrations (MICs) of the antifungal agents were determined both visually and automatically on an ATB Expression Bacteriology Analyzer (bioMérieux, La Balme-les Grottes, France). The quality control strains were *Candida parapsilosis* (ATCC 22019) and *Candida krusei* (ATCC 6258). The interpretative criteria for susceptibility and breakpoints for antifungal drugs were referenced as described by the Clinical and Laboratory Standards Institute M27-A3 microbroth dilution method.

### Molecular Mechanism of Azole Resistance *Candida* Isolates

The *ERG11* and *ERG3* genes were amplified by PCR and sequenced, using the following primers: *ERG11* F: 5'-GTTTTCTACTGGATCCCATG-3', *ERG11* R: 5'-TACATCTGTGTCTACCACC-3'; and *ERG3* F: 5'-ATGGATATCGTACTAGAAATTTGTG-3', *ERG3* R: 5'-TCATTGTTCAACATATTCTCTATCC-3'. The primers were synthesized by Shanghai Sangon Biotech Co., Ltd. Genomic DNA was extracted using the UNIQ-10 Column yeast Plasmid Preps Kit (Sangon Biotech Co., Ltd., Shanghai, China) to be a template for PCR amplification. The amplification program was as follows: denaturation at 94°C, followed by 35 cycles of 30 s at 94°C, 90 s at 50°C for annealing, and 90 s at 72°C for elongation, and by a final elongation step of 8 min at 72°C. The PCR products were semi-quantified by agarose gel electrophoresis and used as templates for sequencing (Sangon Biotech Co., Ltd., Shanghai, China). The amino acid sequences of the genes encoding *ERG11* and *ERG3* were deduced from the nucleotide sequences and then analyzed using the MegAlign software (DNAStar, Inc., Lasergene, Madison, WI, USA). The full nucleotide sequence of the *ERG11* gene of *C. tropicalis* ATCC 750 (GenBank accession number: XM_002550939.1) was used as the reference sequence. The full nucleotide sequence of the *ERG3* gene of *C. tropicalis* MYA-3404 (GenBank accession number: XM002550136.1) was used as the reference sequence.

### Statistical Analysis

The data were analyzed using SPSS software version 22 for Windows (SPSS, Chicago, IL, USA). The categorical data were compared using chi-square tests. Statistical significance was determined using two-tailed tests, and *P* < 0.05 was considered statistically significant. We conducted homology modeling of the three-dimensional structure of Erg11p by SWISS-MODEL using 5JLC (http://www.pdb.org) as the template. The protein structures were visualized by PyMOL software (Schrödinger Inc., Portland, OR, USA).

### Ethical Considerations

The protocols of the study were approved by and carried out following the recommendations of the Life Ethics Committee of Anhui Medical University. All subjects gave their written informed consents as per the Declaration of Helsinki.

## Results

### Clinical Distribution Characteristics of Invasive *Candida* Infections

**Figure 1:**
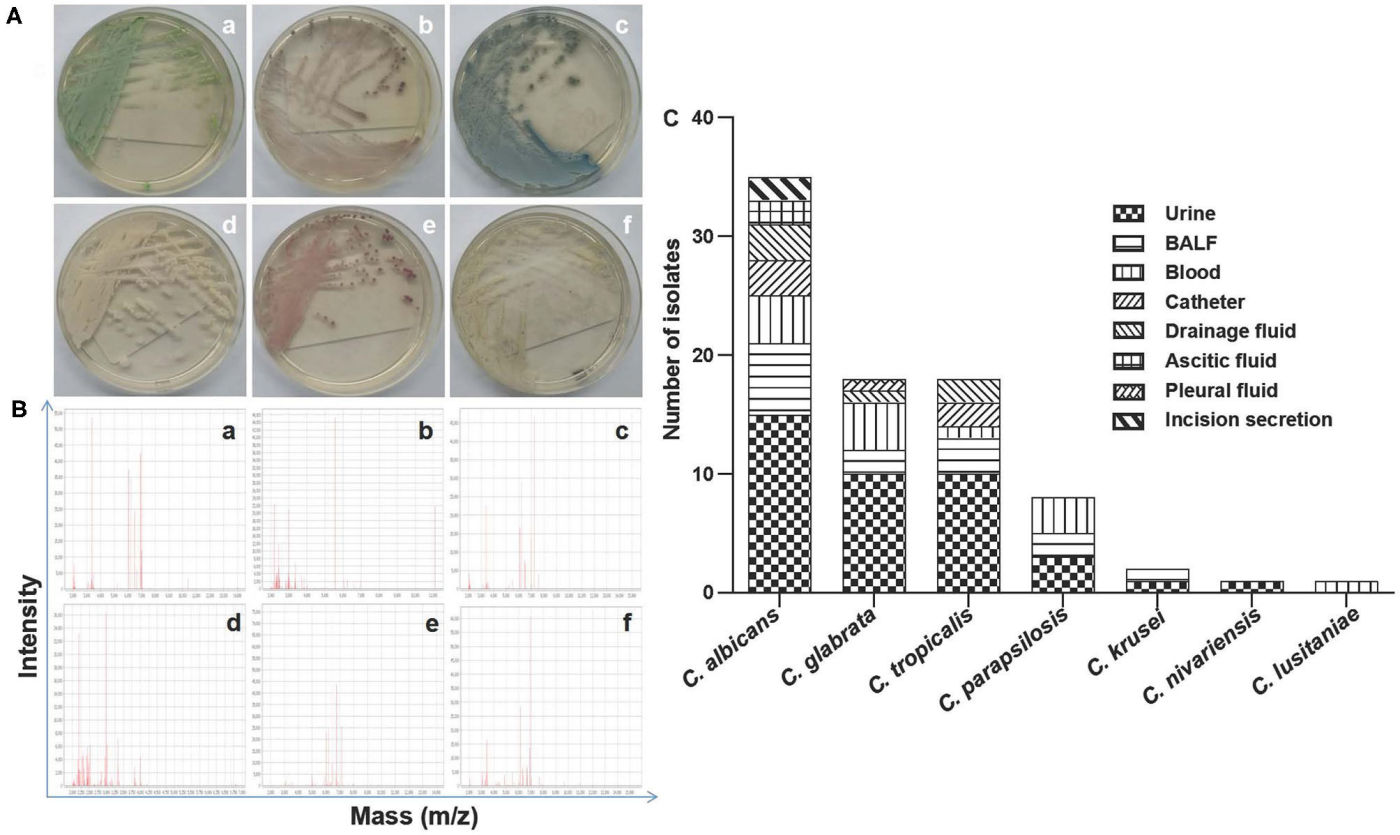
**(A)** Representative culturing macroscopic results of *Candida* spp. (CHROMagar *Candida* chromogenic agar medium and/or Sabouraud Dextrose agar medium). (a–f) *C. albicans, C. glabrata, C. tropicalis, C. parapsilosis, C. krusei*, and *C. lusitaniae*. **(B)** Representative identification information of *Candida* spp. by MALDI-TOF MS. (a–f) *C. albicans, C. glabrata, C. tropicalis, C. parapsilosis, C. krusei*, and *C. lusitaniae*. **(C)** The species distribution and characteristics of specimen sources as indicated from the 83 isolates in the present study. BALF, bronchoalveolar lavage fluid.

During the study period, a total of 115 patients was admitted to the ICU. Among them, 83 patients developed IC, and the rest were neither colonized nor infected with *Candida* species. Totally, 83 *Candida* isolates from the IC individuals were harvested and identified to species level using MALDI-TOF MS, together with macroscopic/microscopic observations of cell morphology (Figures 1A,B). As shown in Figure 1C, the *Candida* species distribution was as follows: *C. albicans* (*n* = 35, 42.17%), *C. glabrata* (*n* = 18, 21.69%), *C. tropicalis* (*n* = 18, 21.69%), *C. parapsilosis* (*n* = 8, 9.64%), *C. krusei* (*n* = 2, 2.41%), *C. lusitaniae* (*n* = 1, 1.20%), *C. nivariensis* (*n* = 1, 1.20%). Notably, *C. albicans* was the predominant species isolated from ICU patients with invasive *Candida* infections; for the non-*C. albicans Candida* (NCAC), both *C. glabrata* and *C. tropicalis* were the major pathogens for those patients. The specimens were of different sources. Nearly half of invasive *Candida* isolates (48.19%) were recovered from urine, followed by BALF (16.87%), blood (15.66%), drainage fluid (7.23%), catheter (6.02%), ascites (2.41%), incision secretion (2.41%), and pleural fluid (1.20%) (Figure 1C).

### Antifungal Susceptibility Patterns

As summarized in Table 1, common *Candida* species were shown highly *in vitro* sensitive to amphotericin B and 5-fluorocytosine (5-FC), though *C. albicans* revealed 97.40% of sensitivity to 5-FC. All *C. albicans* isolates were susceptible to both fluconazole and voriconazole, as were *C. glabrata* and *C. parapsilosis* isolates. Additionally, no itraconazole-resistant *C. albicans* strains were observed in our study, and 94.40% of *C. glabrata* isolates and 87.50% of *C. parapsilosis* isolates were itraconazole-sensitive. It is worth noting that the *C. tropicalis* isolates showed relatively low sensitivity to fluconazole, voriconazole and itraconazole (55.60, 61.10 and 33.30%, respectively) (Table 1). Other rare *Candida* species, except *C. krusei*, showed 100% susceptibility to the five types of antifungal agents.

**Table 1:** *In vitro* effects of antifungal drugs on the isolated strains of *Candida* species.

**Species**	**Drugs**	**MIC (μg/mL)**			**Sensitivity**
		**Range**	**MIC50**	**MIC90**	**(%)**
*C. albicans* (*n* = 35)					
	Fluconazole	1.00–4.00	1.00	1.00	100.00
	Voriconazole	0.06–0.12	0.06	0.06	100.00
	Itraconazole	0.12–0.13	0.13	0.13	100.00
	Amphotericin B	0.50–0.50	0.50	0.50	100.00
	5-fluorocytosine	4.00–16.00	4.00	4.00	97.40
*C. glabrata* (*n* = 18)					
	Fluconazole	1.00–8.00	1.00	2.00	100.00
	Voriconazole	0.06–0.13	0.06	0.13	100.00
	Itraconazole	0.12–0.25	0.13	0.13	94.40
	Amphotericin B	0.50–0.50	0.50	0.50	100.00
	5-fluorocytosine	4.00–4.00	4.00	4.00	100.00
*C. tropicalis* (*n* = 18)					
	Fluconazole	1.00–128.00	2.00	128.00	55.60
	Voriconazole	0.06–8.00	0.25	8.00	61.10
	Itraconazole	0.12–4.00	0.25	4.00	33.30
	Amphotericin B	0.50–0.50	0.50	0.50	100.00
	5-fluorocytosine	4.00–4.00	4.00	4.00	100.00
*C. parapsilosis* (*n* = 8)					
	Fluconazole	1.00–2.00	1.00	2.00	100.00
	Voriconazole	0.06–1.00	0.06	1.00	100.00
	Itraconazole	0.12–0.25	0.13	0.25	87.50
	Amphotericin B	0.50–0.50	0.50	0.50	100.00
	5-fluorocytosine	4.00–4.00	4.00	4.00	100.00
*C. krusei* (*n* = 2)					
	Fluconazole	/	/	/	0.00
	Voriconazole	0.06–0.06	0.06	0.06	100.00
	Itraconazole	/	/	/	0.00
	Amphotericin B	0.50–0.50	0.50	0.50	100.00
	5-fluorocytosine	4.00–4.00	4.00	4.00	100.00
Others (*n* = 2)^*^					
	Fluconazole	1.00–2.00	1.00	2.00	100.00
	Voriconazole	0.06–0.06	0.06	0.06	100.00
	Itraconazole	0.12–0.13	0.13	0.13	100.00
	Amphotericin B	0.50–0.50	0.50	0.50	100.00
	5-fluorocytosine	4.00–4.00	4.00	4.00	100.00

* *Others: one isolate of C. lusitaniae and one isolate of C. nivariensis*.

*/, Intrinsically resistant*.

**Figure 2:**
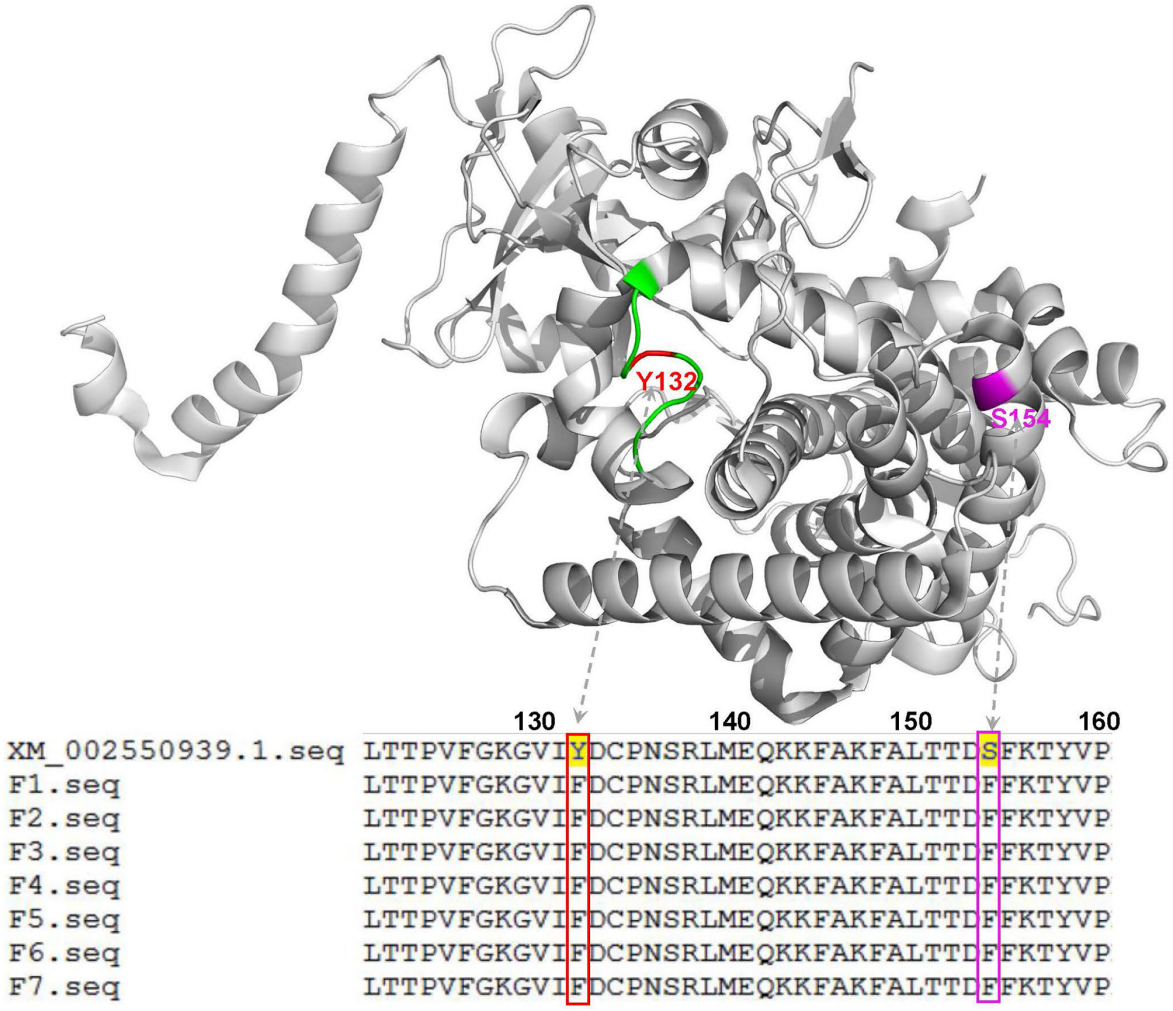
Three-dimensional model of the ERG11 enzyme of *C. tropicalis* using 5JLC (http://www.pdb.org) as the template to analyze the indicated gene sequences with mutations in this study. Green, the BC loop; red, the mutation site of Y132F; magenta, the mutation site of S154F. XM_002550939.1, the indicated gene sequence provided by GenBank; F1-F7, the 7 isolates of *C. tropicalis* with azole resistance.

To further explore the molecular mechanisms of *C. tropicalis* resistance to azoles, a total of seven azole-resistant *C. tropicalis* were obtained, and their *ERG11* and *ERG3* genes were determined by PCR and sequencing. Through gel electrophoresis analysis, it was confirmed that the PCR products of the target genes were consistent with expectations. In the sequencing analysis, the full lengths of nucleotide sequences of *ERG11* (GenBank accession number: XM_002550939.1) and *ERG3* (GenBank accession number: XM002550136.1) in *C. tropicalis* were used as reference sequences. As shown in Figure 2, with the assistance of a three-dimensional model of ERG11 enzyme of *C. tropicalis* using 5JLC (http://www.pdb.org), our sequencing data of *ERG11* revealed two single missense mutations (A395T and C461T) in all the seven azole-resistant isolates of *C. tropicalis*, leading to the amino acid substitutions of Y132F and S154F, respectively (GenBank accession number: MZ703041). In addition, all the seven isolates possessed two synonymous mutations (T225C and G264A) in *ERG11*. Meanwhile, *ERG3* sequencing data exhibited only one synonymous mutation (G366A) in three out of the seven azole-resistant *C. tropicalis* (GenBank accession number: MZ703040).

### Clinical Characteristics and Outcomes of Patients With Invasive *Candida* Infections

In this study, a similar sex distribution pattern was observed among the 83 patients with IC, namely 53.01% (44/83) of males vs. 46.09% (39/83) of females. The most commonly isolated invasive fungi from males were *C. albicans* (47.73%, 21/44) and *C. tropicalis* (22.73%, 10/44). However, *C. glabrata* (28.21%, 11/39) and *C. albicans* (35.90%, 14/39) were the primary invasive *Candida* from females. It's worth noting that two rare *Candida* isolates of *C. nigeriana* and *C. lusitaniae* were identified within females (Table 2). Next, we further observed that the patient age posed a positive impact on the fungal infection rates in our study. As summarized in Table 2, the most susceptible age was over 50 years among ICU patients with IC, accounting for 80.72% (67/83). Comparatively, significant difference was observed between patients with and without IC in both the age groups of over 65 years (*P* < 0.001) and 15–49 years (*P* < 0.001). Moreover, the length of stay in the ICU was also positively correlated with the rate of *Candida* infections. More than half of the infections occurred in ICU patients hospitalized for more than 14 days (65.06%, 54/83). Importantly, among the various relevant risk factors which significantly influence the incidence of *Candida* infections, tracheotomy, diabetes and concomitant bacterial infection were found to be statistically significant (*P* < 0.05, Table 2). Whereas, the rest risk factors did not show significant differences (*P* > 0.05) including urinary catheterization, tracheal intubation, ventilator support, arteriovenous cannulation, surgery, hemodialysis/peritoneal dialysis, immunosuppressive treatment, cancer, and cirrhosis. When compared with IC patients in the ICU, the outcomes of the ones without IC were significantly improved (*P* < 0.001).

**Table 2:** Demographic and clinical characteristics of ICU patients (*n* = 115).

**Clinical characteristics**	**Patient positive for *Candida***	**Patient negative for *Candida***	***P***
	***n* = 83 (%)**	***n* = 32 (%)**	
**Gender**			
Male	44 (53.01)	23 (71.87)	0.066
Female	39 (46.09)	9 (28.13)	
**Age**			
0~14	2 (2.41)	0 (0)	0.376
15~49	14 (16.87)	20 (62.50)	<0.001
50~65	27 (32.53)	6 (18.75)	0.143
>65	40 (48.19)	4 (12.50)	<0.001
**ICU length of stay**			
<7 days	5 (6.02)	9 (28.13)	0.003
7~14 days	23 (27.71)	12 (37.50)	0.426
>14 days	54 (65.06)	11 (34.38)	0.003
**Risk factors**			
*Invasive intervention/procedure*			
Urinary catheters	70 (84.34)	26 (81.25)	0.690
Tracheal intubation	69 (83.13)	29 (90.63)	0.471
Ventilator support	71 (85.54)	30 (93.75)	0.374
Arteriovenous cannulation	60 (72.29)	22 (68.75)	0.707
Tracheotomy	50 (60.24)	12 (37.50)	0.028
Surgery	34 (40.96)	18 (56.25)	0.140
Hemodialysis/peritoneal dialysis	14 (16.87)	6 (18.75)	0.811
*Immunosuppressive state*			
Immunosuppressive treatment	15 (18.07)	9 (28.13)	0.235
*Underlying disease*			
Diabetes	25 (30.12)	2 (6.25)	0.007
Cancer	15 (18.07)	2 (6.25)	0.191
Cirrhosis	3 (3.61)	2 (6.25)	0.912
Concomitant Candida infection^*^	34 (40.96)	0	<0.001
*Concomitant bacterial infection* ^†^	60 (72.29)	13 (40.63)	0.002
**Outcome**			
Improvement	36 (43.37)	31 (96.88)	<0.001
Deterioration	31 (37.35)	1 (3.13)	<0.001
Death	6 (7.23)	0 (0)	0.274

* *The same Candida spp. isolated in more than one site*.

† *Patients co-infected with Candida spp. and bacteria*.

With the increase of clinical antifungal resistance in *Candida* infections, special attention should be drawn to the azole-resistant *C. tropicalis* in the present study. For the seven confirmed azole-resistant *C. tropicalis* isolates we obtained, their host patients were 46–81 years old, with the mean age of 59.43 years. And the gender ratio (male to female) was 2.5 to 1. In general, the distribution of underling diseases in the seven patients with azole-resistant isolates was as follows: heart disease (*n* = 4), burns (*n* = 1), cancer (*n* = 1), and liver transplantation (*n* = 1). Four cases were diagnosed with septicemia/septic shock, and the other three cases with pulmonary infections (Table 3). In addition, cultures from four patients were positive for azole-resistant *C. tropicalis* in more than one site. Notably, three of the seven patients were azole naive, and the other four patients had previously received fluconazole/voriconazole treatment. All the *C. tropicalis* isolates from patients with azole-resistant invasive *Candida* shared the resistance mechanisms of Y132F and S154F. The outcomes of two patients receiving fluconazole or voriconazole monotherapy ended up with clinical deterioration. While for the remaining five patients, voriconazole monotherapy was switched into a combination antibiotic therapy with more than one antifungal agent including a class of antifungal compounds, *i.e*., posaconazole, caspofungin, or both. Four out of the five patients eventually significantly restrained the infections (Table 3).

**Table 3:** Characteristics of patients with azole-resistant *C. tropicalis*.

**Patient age/gender**	**Underlying disease**	**Disease**	**No. of positive cultures**	**Resistance mechanism**	**Infection sites**	Prior azole treatment (duration)^*^	**Treatment^†^**	**Outcome^*††*^**
67/f	Malignant neoplasm of sigmoid colon	Septic shock	7	Y132F, S154F	1	N/A	FCZ	Deterioration
54/f	Type 2 diabetes mellitus; tricuspid insufficiency; mitral valve replacement	Pulmonary infection	6	Y132F, S154F	2	N/A	VCZ	Deterioration
54/f	Mitral insufficiency	Pulmonary infection; septicemia	18	Y132F, S154F	5	N/A	VCZ, CAS	Deterioration
46/m	Burns	Pulmonary infection; septic shock	3	Y132F, S154F	1	FCZ	VCZ, CAS	Control of infection
81/m	Coronary atherosclerotic heart disease	Pulmonary infection; asthma	7	Y132F, S154F	2	VCZ	VCZ, CAS	Control of infection
52/f	Liver transplantation	Pulmonary infection; septicemia	10	Y132F, S154F	4	VCZ	VCZ, CAS, POS	Control of infection
62/f	Valvular heart disease	Pulmonary infection	8	Y132F, S154F	1	VCZ	VCZ, CAS, POS	Control of infection

*f, female; m, male; N/A, not applicable; VCZ, voriconazole; CAS, caspofungin; FCZ, fluconazole; POS, posaconazole*.

* *Azole treatment before identification of resistant isolates in laboratory*.

† *Treatment after identification of resistant isolates in laboratory*.

†† *Deterioration, persistence or progression of azole-resistant C. tropicalis infection, or patients were dead or discharged with voluntary withdrawal of treatment with unknown reasons; Control of infection, clearance of azole-resistant C. tropicalis (approximately within 1~2 weeks) during the ICU stay*.

## Discussion

IC is increasingly involved with hospital-acquired infections. It usually gives rise to relatively high morbidity and mortality, especially among the acute or severe patients in the ICUs. These ICU patients are often treated with clinically invasive interventions, broad-spectrum antibiotics and/or hormonotherapy. Consequently, the frequent invasive interventions will be able to compromise the mucocutaneous barrier protection. Extensive utilization of multiple antifungals will inevitably raise drug resistance. And hormonotherapy is prone to host immunosuppression, increasing the infection risk of IC (2). It is essential to early diagnose the disease and immediately give appropriate antifungals to treat ICU patients with IC (11). The exogenous source of hospital-borne infections in the ICUs may be person-to-person contact, contaminated equipment or building services in hospital (5). Therefore, reducing interpersonal cross-infections and microbial colonization of equipment/devices is pivotal to prevent candidiasis in ICU patients.

Since the coronavirus disease 2019 outbreak during the study period and the implementation of the hierarchical medical system in China (12) that classifies distinct degrees of diseases according to doctors' diagnosis, medical institutions at all levels provide continuing medical services, medical resources are allocated based on demands in recent years, more acute and severe patients such as critically ill patients with diabetes and (solid organ) transplantations have preferentially been admitted to the ICU in our Province-level tertiary hospital, seemingly resulting in a relatively high proportion of the infections. This may not be the case for the actual average infection rate. Therefore, no specific analysis was directly or arbitrarily conducted in the present study on the issue of the overall infection rates. Although *C. albicans* was earlier the dominant pathogen accounting for two-thirds of the infections, an increasing number of NCAC can currently be identified and responsible for almost 50% of the infections (13). Similarly, our data revealed that *C. albicans* was still the principal pathogen causing invasive fungal infections in our ICU patients, while *C. glabrata* and *C. tropicalis* were the two major NCAC of invasive candidiasis. Nevertheless, studies reported that *C. parapsilosis* was the most common NCAC for invasive candidiasis in ICU patients in other parts of China (14). Various risk factors, alone or in combination, influence the frequency and nature of *Candida* infections, including specimen source, age, length of hospital stay, underlying diseases, *etc*. (11, 15, 16). In our study, urine, BALF, and blood were the main sources of specimens. To be noted, the majority of *Candida* spp. were harvested from urine, which presented a different source pattern with other studies (17, 18). That difference is likely attributable to the seasonal, regional and environmental preferences of the fungi including genus *Candida*. Furthermore, the patient's underlying disease (*e.g*., diabetes) and immunosuppressive state (*e.g*., with renal transplantation) may primarily contribute to the relatively high incidence of *Candida* urinary tract infections in this study.

The patients with IC involved were nearly elderly and experienced relatively long length of hospital stay, more than half were over 50 years old and with >14 days of ICU stay. Amidst the indicated risk factors, tracheotomy, diabetes mellitus and concomitant bacterial infection were highly indicative of the occurrence of invasive candidiasis in ICU patients in the present study. Invasive procedure, *e.g*., tracheotomy, is regularly a key factor entailing an increased chance of *Candida* colonization/infection in ICU patients. Previous studies showed that diabetes mellitus was able to predispose one to systemic candidiasis due to several factors, among which the progression of microvascular disease remarkably contributed to the mechanism of lowered host defense, and the diabetic vasculopathy exacerbating hypoperfusion and hyperglycemia and likely leading to neutrophil and lymphocyte dysfunctions with impaired opsonization (19, 20). The clinical significance of polymicrobial interactions, particularly those between bacteria and fungi with high pathogenic potentials, remains largely underestimated, although several studies reported that bacterial infection might be a risk factor for disseminated candidiasis. It is necessary to raise its awareness in the treatment and management of immunocompromised individuals (21, 22). The specific mechanisms of interaction between bacterial and fungal infections require further investigations.

The antifungal agents can be divided into different categories, including azoles, polyenes, fluoropyrimidine analogs, echinocandins, morpholines, allylamines, thiocarbamates, and 5-FC (23). In this study, we analyzed the sensitivity of *Candida* spp. to five antifungal drugs commonly used in clinic. Our results demonstrated that all of the strains were sensitive to amphotericin B, and over 90% of them exhibited susceptibility to 5-FC (only 2.60% of *C. albicans* were resistant). Other researchers declare that the pathogenic spectrum of *Candida* infections has changed over the past decade, with a gradual shift from *C. albicans* to NCAC that may be less susceptible to azoles (24). The *Candida* spp. in this study presented a significant diversity in sensitivity to the individual members of azoles. Briefly, *C. tropicalis* showed relatively low sensitivity to fluconazole, voriconazole and itraconazole; *C. albicans* were all sensitive to all the members of azoles; *C. glabrata, C. tropicalis, C. parapsilosis*, and *C. krusei* were comparatively less susceptible to itraconazole. Currently, itraconazole has only oral formulations available and is generally reserved for patients with mucosal candidiasis or outpatient use, nevertheless itraconazole is not well recommended to treat patients with IC which likely prefer its intravenous formulations if supported by large well-controlled clinical trials (25, 26). Therefore, the drug sensitivity test of itraconazole conducted in this study depicted its *in vitro* efficacy against *Candida* spp., which potentially provides knowledge for possible azole cross-resistance, concomitant *Candida* infections, and better antifungal drug designs in future. These observations produced similar drug sensitivity patterns with other studies, but with slight differences (27, 28); for instance, no fluconazole-resistant *C. glabrata* was observed in the present study, which probably is the current drug resistance feature of our local region, though this difference may also be caused by the limited sample size. Hence, our data indicate that there might be a species-dependent susceptibility of invasive fungi to azoles. Frequent use of fluconazole in the treatment of IC may be a cause of the increased antifungal resistance in ICU patients. These findings have enormous potential in guiding the use of antifungal drugs for ICU patients with IC.

The mechanisms of *Candida* resistance include altered drug affinity and/or target abundance, reduced drug uptake via efflux pumps, and formation of biofilms which resist drug action (29, 30). Certain *C. tropicalis* we found exhibited a high-level resistance to fluconazole, itraconazole, and voriconazole, and even presented a cross-resistance effect. *C. tropicalis* drug resistance mechanism is closely associated with gene mutations (mainly *ERG11* and *ERG3*) (31). Our findings further unveiled double homozygous mutations of A395T and C461T in *ERG11* gene among all azole-resistant *C. tropicalis* strains, resulting in the amino acid substitutions of Y132C and S154C in ERG11. These mutations are believed to attenuate the affinity of ERG11 for azoles and give rise to azole resistance (32, 33). Several studies reported *in vivo* synergistic effects of azole plus caspofungin on multidrug-resistant *C. glabrata* and *C. albicans* (34, 35). Notably, co-administration of posaconazole plus echinocandins was well tolerated without changing the pharmacokinetics of either agent (36). Similarly, in the present study, despite other factors that may affect the outcome of treatment, two of the three patients with deterioration were treated only with azole monotherapy, while the other four patients with controlled infection were treated with the combination of azole plus echinocandins antifungals. However, due to limited case size, whether the combination therapy could be an alternative to monotherapy for patients with azole-resistant *C. tropicalis* needs to be further addressed.

## Conclusions

In summary, this study reported the *Candida* species distribution and their antifungal sensitivities, and clinical characteristics of ICU patients with invasive *Candida* infections in central part of China. Our results showed that the age, length of hospitalization, tracheotomy, diabetes mellitus as well as concomitant bacterial infection could be considered as the main risk factors for candidiasis. The polymorphism of the *ERG11* gene in *C. tropicalis* may be closely associated with azole resistance.

## Data Availability Statement

The datasets presented in this study can be found in online repositories. The names of the repository/repositories and accession number(s) can be found at: https://www.ncbi.nlm.nih.gov/, MZ703041; https://www.ncbi.nlm.nih.gov/, MZ703040.

## Ethics Statement

The studies involving human participants were reviewed and approved by the Life Ethics Committee of Anhui Medical University. Written informed consent to participate in this study was provided by the participants' legal guardian/next of kin.

## Author Contributions

BW, YH, and JX conceived and designed the experiments. BW, JX, YW, and YL designed the research protocol and performed the experiments. BW, JX, and MZ performed data acquisition and analysis. BW, XH, FL, YL, MZ, YH, and JX contributed to the interpretation of results and assisted in writing the manuscript. All authors read and approved the final manuscript.

## Funding

This study was supported by the National Natural Science Foundation of China (81601446) (BW) and the Natural Science Foundation of Anhui Province (1708085QH210) (BW). The funders had no role in the study design, data collection, and analysis, decision to publish, or preparation of the manuscript.

## Conflict of Interest

The authors declare that the research was conducted in the absence of any commercial or financial relationships that could be construed as a potential conflict of interest.

## Publisher's Note

All claims expressed in this article are solely those of the authors and do not necessarily represent those of their affiliated organizations, or those of the publisher, the editors and the reviewers. Any product that may be evaluated in this article, or claim that may be made by its manufacturer, is not guaranteed or endorsed by the publisher.

## Abbreviations

ICU: intensive care unit
IC: invasive candidiasis
ERG11: 14-α-sterol demethylase
ERG3: sterol δ5,6-desaturase
BALF: bronchoalveolar lavage fluid
MALDI-TOF MS: matrix-assisted laser desorption ionization-time of flight mass spectrometry.
